# Supplementary material for: Effect of erythromycin residuals in food on the development of resistance in Streptococcus pneumoniae: an in vivo study in Galleria mellonella
Source: PeerJ. 2024 May 28;12:e17463. doi: 10.7717/peerj.17463 (PMC11141549; doi:10.7717/peerj.17463)
Supplement: Supplemental Information 1 [file peerj-12-17463-s001.docx]

**Supplementary Material**

**Supplementary Table 1.** Weight of the batch 2 larvae used in the experiment

| **Condition** | **Weight of the larvae used in experiment, mg** | | | | | | | | | | | | | | | | | | | | **Average weight** |
| --- | --- | --- | --- | --- | --- | --- | --- | --- | --- | --- | --- | --- | --- | --- | --- | --- | --- | --- | --- | --- | --- |
| PBS (ctrl) | 329 | 315 | 371 | 352 | 346 | 351 | 363 | 382 | 350 | 356 | 310 | 348 | 371 | 352 | 375 | 351 | 364 | 305 | 354 | 357 | 350.1 |
| 1ADI EMA ERM | 348 | 362 | 379 | 353 | 312 | 337 | 344 | 353 | 361 | 348 | 348 | 362 | 369 | 376 | 345 | 339 | 308 | 355 | 360 | 347 | 350.3 |
| 10ADI EMA ERM | 367 | 355 | 360 | 314 | 360 | 373 | 352 | 349 | 351 | 357 | 368 | 357 | 305 | 355 | 361 | 375 | 345 | 304 | 365 | 340 | 350.65 |
| 0.1ADI WHO ERM | 359 | 312 | 358 | 371 | 350 | 375 | 351 | 364 | 309 | 364 | 334 | 315 | 373 | 365 | 346 | 361 | 363 | 372 | 355 | 306 | 350.15 |
| 1ADI WHO ERM | 346 | 323 | 362 | 359 | 371 | 345 | 339 | 308 | 352 | 356 | 370 | 360 | 357 | 310 | 361 | 374 | 352 | 348 | 351 | 353 | 349.85 |

**Supplementary Table 2a.** Species identity of colonies isolated on non erythromycin plates confirmed by MALDI-TOF (Batch 2)

| **Condition** | **Day** | № of ***S. pneumoniae*** colonies | № of **non- *S. pneumoniae*** colonies | **Non-*S. pneumoniae* ID** |
| --- | --- | --- | --- | --- |
| PBS(ctrl) | 1 | 3 | 2 | *E. casseliflavus, S. avidinii* |
|  | 2 | 1 | 3 | *S. hominis, E. gallinarum* |
|  | 3 | 2 | 2 | *E. gallinarum, S. avidinii* |
|  | 4 | 2 | 1 | *E. casseliflavus* |
|  | 5 | 1 | 2 | *L. agilis, S. sciuri* |
|  | 6 | 2 | 2 | *E. gallinarum* |
|  |  |  |  |  |
| 1ADI EMA ERM | 1 | 1 | 2 | *E. casseliflavus* |
|  | 2 | 3 | 1 | *S. klosii* |
|  | 3 | 2 | 1 | *E. casseliflavus* |
|  | 4 | 1 | 3 | *E. gallinarum, C. freundii* |
|  | 5 | 2 | 1 | *E. casseliflavus* |
|  | 6 | 1 | 2 | *S. klosii, E. mundtii* |
|  |  |  |  |  |
| 10ADI EMA ERM | 1 | 2 | 2 | *S.marcescens, E.casseliflavus* |
|  | 2 | 1 | 2 | *E. gallinarum, E. mundtii* |
|  | 3 | 1 | 3 | *B. thailandensis, E. casseliflavus* |
|  | 4 | 2 | 1 | *S. klosii* |
|  | 5 | 1 | 2 | *E. mundtii, E. gallinarum* |
|  | 6 | 1 | 1 | *L. agilis* |
|  |  |  |  |  |
| 0.1ADI WHO ERM | 1 | 3 | 1 | *S. avidinii* |
|  | 2 | 1 | 2 | *E. gallinarum,  S. klosii* |
|  | 3 | 2 | 1 | *S. hominis* |
|  | 4 | 2 | 3 | *E. casseliflavus,  E. gallinarum* |
|  | 5 | 1 | 1 | *E. mundtii* |
|  | 6 | 2 | 1 | *E. casseliflavus* |
|  |  |  |  |  |
| 1ADI WHO ERM | 1 | 2 | 1 | *E. mundtii* |
|  | 2 | 3 | 2 | *S. hominis,  E. casseliflavus* |
|  | 3 | 1 | 2 | *E. gallinarum* |
|  | 4 | 1 | 3 | *E. casseliflavus,  E. mundtii* |
|  | 5 | 2 | 1 | *S. avidinii* |
|  | 6 | 1 | 2 | *A. encheleia,  E. mundtii* |

**Supplementary Table 2b.** Species identity of colonies isolated on erythromycin plates confirmed by MALDI-TOF (Batch 2)

| **Condition** | **Day** | № of ***S. pneumoniae*** colonies | № of **non- *S. pneumoniae*** colonies | **Non-*S. pneumoniae* ID** |
| --- | --- | --- | --- | --- |
| PBS(ctrl) | 1 | 0 | 0 |  |
|  | 2 | 0 | 0 |  |
|  | 3 | 0 | 0 |  |
|  | 4 | 0 | 0 |  |
|  | 5 | 0 | 0 |  |
|  | 6 | 0 | 0 |  |
|  |  |  |  |  |
| 1ADI EMA ERM | 1 | 4 | 0 |  |
|  | 2 | 0 | 0 |  |
|  | 3 | 0 | 0 |  |
|  | 4 | 4 | 0 |  |
|  | 5 | 0 | 0 |  |
|  | 6 | 0 | 0 |  |
|  |  |  |  |  |
| 10ADI EMA ERM | 1 | 0 | 0 |  |
|  | 2 | 0 | 0 |  |
|  | 3 | 0 | 0 |  |
|  | 4 | 0 | 0 |  |
|  | 5 | 0 | 0 |  |
|  | 6 | 0 | 0 |  |
|  |  |  |  |  |
| 0.1ADI WHO ERM | 1 | 0 | 0 |  |
|  | 2 | 0 | 0 |  |
|  | 3 | 0 | 0 |  |
|  | 4 | 0 | 0 |  |
|  | 5 | 0 | 0 |  |
|  | 6 | 0 | 0 |  |
|  |  |  |  |  |
| 1ADI WHO ERM | 1 | 0 | 1 | *Enterococcus casseliflavus* |
|  | 2 | 0 | 0 |  |
|  | 3 | 0 | 0 |  |
|  | 4 | 4 | 0 |  |
|  | 5 | 0 | 0 |  |
|  | 6 | 0 | 0 |  |

**Supplementary Table 3.** Identification of **(a)** virulence genes using Vfdb database and **(b)** antimicrobial resistance genes using card database

**(a)**

| **VfdB** |  |  |  |  |  |  |  |  |  |  |  |  |  |  |  |  |
| --- | --- | --- | --- | --- | --- | --- | --- | --- | --- | --- | --- | --- | --- | --- | --- | --- |
| Genome Id | *cbpD* | *cbpG* | *cps4A* | *cps4B* | *cps4C* | *cps4D* | *hysA* | *lytA* | *lytB* | *lytC* | *nanB* | *pavA* | *pce* | *pfbA* | *ply* | *psaA* |
| SP_0603A1_SC_E14587_lib691262_10231 | 100 | 99.77 | 99.79 | 99.86 | 99.86 | 100 | 100 | 97.07;100.00 | 100 | 100 | 100 | 100 | 100 | 100 | 100 | 100 |
| SP_1403_1B_E14589_lib691264_10231 | 100 | 99.77 | 99.79 | 99.86 | 99.86 | 100 | 100 | 97.07;100.00 | 100 | 100 | 100 | 100 | 100 | 100 | 100 | 100 |
| SP_1403_1C_E14586_lib691261_10231 | 100 | 99.77 | 99.79 | 99.86 | 99.86 | 100 | 100 | 100.00;97.07 | 100 | 100 | 100 | 100 | 100 | 100 | 100 | 100 |
| SP_2802_B_E14588_lib691263_10231 | 100 | 99.77 | 99.79 | 99.86 | 99.86 | 100 | 100 | 100.00;97.07 | 100 | 100 | 100 | 100 | 100 | 100 | 100 | 100 |
| SP_ATCC_YB_lib707253_10264 | 100 | 99.77 | 99.79 | 99.86 | 99.86 | 100 | 100 | 97.07;100.00 | 100 | 100 | 100 | 100 | 100 | 100 | 100 | 100 |
| SP_ctrl_B2_lib717407_10294 | 100 | 99.77 | 99.79 | 99.86 | 99.86 | 100 | 100 | 97.07;100.00 | 100 | 100 | 100 | 100 | 100 | 100 | 100 | 100 |
| SP_ctrl_B4_lib717408_10294 | 100 | 99.77 | 99.79 | 99.86 | 99.86 | 100 | 100 | 97.07;100.00 | 100 | 100 | 100 | 100 | 100 | 100 | 100 | 100 |

**(b)**

| **CARD** |  |  |  |  |  |
| --- | --- | --- | --- | --- | --- |
| Genome Id | *RlmA(II)* | *patA* | *patB* | *pmrA* | *vanRC* |
| NG-31926_SP_0603A1_SC_E14587_lib691262_10231.fna | 100 | 100 | 100 | 100 | . |
| NG-31926_SP_1403_1B_E14589_lib691264_10231.fna | 100 | 100 | 100 | 100 | . |
| NG-31926_SP_1403_1C_E14586_lib691261_10231.fna | 100 | 100 | 100 | 100 | . |
| NG-31926_SP_2802_B_E14588_lib691263_10231.fna | 100 | 100 | 100 | 100 | 99.86 |
| NG-31926_SP_ATCC_YB_lib707253_10264.fna | 100 | 100 | 100 | 100 | . |
| NG-34157_SP_ctrl_B2_lib717407_10294.fna | 100 | 100 | 100 | 100 | . |
| NG-34157_SP_ctrl_B4_lib717408_10294.fna | 100 | 100 | 100 | 100 | . |

**Supplementary Table 4.** All SNPs found for *S. pneumoniae* isolates

| **Gene Product** | **CDS** | **2802b** | **1403b** | **1403c** | **0303c** | **Ctrl_B2** | **Ctrl_B4** | **ATCC 49619** |
| --- | --- | --- | --- | --- | --- | --- | --- | --- |
| Hypothetical Protein | CDS: OPMNIGBM_00355, Gene: OPMNIGBM_00355 | #N/A | #N/A | #N/A | X:OPMNIGBM_00355:c.888A>C | #N/A | #N/A | #N/A |
| Hypothetical Protein | Gene: OPMNIGBM_00536, CDS: OPMNIGBM_00536 | #N/A | #N/A | #N/A | X:OPMNIGBM_00536:p.Arg77Trp | #N/A | #N/A | #N/A |
| Tyrosine recombinase | CDS: xerS, Gene: xerS | #N/A | #N/A | #N/A | X:OPMNIGBM_00645:p.Val324Leu | #N/A | #N/A | #N/A |
| 1,4-alpha-glucan branching enzyme | CDS: glgB, Gene: glgB | X:OPMNIGBM_00661:p.Val194Gly | X:OPMNIGBM_00661:p.Val194Gly | X:OPMNIGBM_00661:p.Val194Gly | X:OPMNIGBM_00661:p.Val194Gly | X:OPMNIGBM_00661:p.Val194Gly | X:OPMNIGBM_00661:p.Val194Gly | #N/A |
| ISL3 family transposase ISSpn14 | CDS: OPMNIGBM_00680, Gene: OPMNIGBM_00680 | X:OPMNIGBM_00680:c.117A>G | X:OPMNIGBM_00680:p.Thr45Ile | X:OPMNIGBM_00680:c.183T>C | X:OPMNIGBM_00680:c.183T>C | X:OPMNIGBM_00680:c.117A>G | X:OPMNIGBM_00680:c.117A>G | X:OPMNIGBM_00680:p.Thr45Ile |
| IS630 family transposase ISSpn2 | CDS: OPMNIGBM_00796, Gene: OPMNIGBM_00796 | X:OPMNIGBM_00796:p.Lys59fs | X:OPMNIGBM_00796:p.Lys59fs | X:OPMNIGBM_00796:p.Lys59fs | X:OPMNIGBM_00796:p.Lys59fs | X:OPMNIGBM_00796:p.Lys59fs | X:OPMNIGBM_00796:p.Lys59fs | X:OPMNIGBM_00796:p.Lys59fs |
| IS630 family transposase ISSpn2 | CDS: OPMNIGBM_00797, Gene: OPMNIGBM_00797 | X:OPMNIGBM_00797:p.Val3Ala | X:OPMNIGBM_00797:p.Val3Ala | X:OPMNIGBM_00797:p.Val3Ala | X:OPMNIGBM_00797:p.Val3Ala | X:OPMNIGBM_00797:p.Val3Ala | X:OPMNIGBM_00797:p.Val3Ala | X:OPMNIGBM_00797:p.Val3Ala |
| Hypothetical Protein | CDS: OPMNIGBM_00800, Gene: OPMNIGBM_00800 | #N/A | #N/A | #N/A | X:OPMNIGBM_00800:p.Ser121Gly | #N/A | #N/A | #N/A |
| Putative TrmH family tRNA/rRNA methyltransferase | CDS: OPMNIGBM_00823, Gene: OPMNIGBM_00823 | #N/A | #N/A | #N/A | X:OPMNIGBM_00823:p.Ser203Arg | #N/A | #N/A | #N/A |
| Vitamin B12 import ATP-binding protein BtuD | CDS: btuD_3, Gene: btuD_3 | X:OPMNIGBM_00839:c.708A>C | X:OPMNIGBM_00839:c.708A>C | X:OPMNIGBM_00839:c.708A>C | X:OPMNIGBM_00839:c.708A>C | X:OPMNIGBM_00839:c.708A>C | X:OPMNIGBM_00839:c.708A>C | #N/A |
| Hypothetical Protein | CDS: OPMNIGBM_00924, Gene: OPMNIGBM_00924 | X:OPMNIGBM_00924:c.123G>A | X:OPMNIGBM_00924:c.123G>A | X:OPMNIGBM_00924:c.123G>A | X:OPMNIGBM_00924:c.123G>A | X:OPMNIGBM_00924:c.123G>A | X:OPMNIGBM_00924:c.123G>A |  |
| Hypothetical Protein | CDS: OPMNIGBM_00925, Gene: OPMNIGBM_00925 | #N/A | #N/A | #N/A | X:OPMNIGBM_00925:p.Trp47Leu | #N/A | #N/A | #N/A |
| Ribulose-5-phosphate reductase | CDS: tarJ, Gene: tarJ | X:OPMNIGBM_01124:p.Thr63Met | #N/A | X:OPMNIGBM_01124:p.Thr63Met | X:OPMNIGBM_01124:p.Thr63Met | X:OPMNIGBM_01124:p.Thr63Met | X:OPMNIGBM_01124:p.Thr63Met | #N/A |
| Hypothetical Protein | CDS: OPMNIGBM_01263, Gene: OPMNIGBM_01263 | #N/A | #N/A | #N/A | X:OPMNIGBM_01263:p.Leu8Ser | #N/A | #N/A | #N/A |
| Hypothetical Protein | CDS: OPMNIGBM_01320, Gene: OPMNIGBM_01320 | X:OPMNIGBM_01320:p.Gln1702Pro | X:OPMNIGBM_01320:p.Gln1702Pro | X:OPMNIGBM_01320:p.Gln1702Pro | X:OPMNIGBM_01320:p.Gln1702Pro | X:OPMNIGBM_01320:p.Gln1702Pro | X:OPMNIGBM_01320:p.Gln1702Pro | #N/A |
| Sugar phosphatase YidA | Gene: yidA_2, CDS: yidA_2 | X:OPMNIGBM_01507:p.Pro60Leu | #N/A | X:OPMNIGBM_01507:p.Pro60Leu | X:OPMNIGBM_01507:p.Pro60Leu | X:OPMNIGBM_01507:p.Pro60Leu | X:OPMNIGBM_01507:p.Pro60Leu | #N/A |
| IS3 family transposase ISSpn11 | CDS: OPMNIGBM_01705, Gene: OPMNIGBM_01705 | X:OPMNIGBM_01705:p.Tyr91fs | X:OPMNIGBM_01705:p.His57Tyr | X:OPMNIGBM_01705:p.Ala52Gly | X:OPMNIGBM_01705:p.Thr30Pro | X:OPMNIGBM_01705:p.Thr30Pro | X:OPMNIGBM_01705:p.Thr30Pro | X:OPMNIGBM_01705:p.Thr30Pro |
| ISL3 family transposase IS1167 | CDS: OPMNIGBM_01721, Gene: OPMNIGBM_01721 | X:OPMNIGBM_01721:p.Lys65Glu | X:OPMNIGBM_01721:p.Lys65Glu | X:OPMNIGBM_01721:p.Lys65Glu | X:OPMNIGBM_01721:p.Lys65Glu | X:OPMNIGBM_01721:p.Lys65Glu | X:OPMNIGBM_01721:p.Lys65Glu | X:OPMNIGBM_01721:p.Lys65Glu |
| Hypothetical Protein | Gene: OPMNIGBM_00216, CDS: OPMNIGBM_00216 | #N/A | X:OPMNIGBM_00216:p.Glu159* | #N/A | #N/A | #N/A | #N/A | #N/A |
| IS5 family transposase ISSpn7 | CDS: OPMNIGBM_00292, Gene: OPMNIGBM_00292 | X:OPMNIGBM_00292:p.Ala3Thr | X:OPMNIGBM_00292:p.Ala3Thr | #N/A | #N/A | X:OPMNIGBM_00292:p.Ala3Thr | X:OPMNIGBM_00292:p.Ala3Thr | X:OPMNIGBM_00292:p.Ala3Thr |
| ISL3 family transposase IS1167 | CDS: OPMNIGBM_00678, Gene: OPMNIGBM_00678 | X:OPMNIGBM_00678:p.Asn25Lys | X:OPMNIGBM_00678:c.189T>C | #N/A | #N/A | X:OPMNIGBM_00678:p.Asn25Lys | X:OPMNIGBM_00678:p.Asn25Lys | X:OPMNIGBM_00678:p.Asn25Lys |
| Hypothetical Protein | CDS: OPMNIGBM_00913, Gene: OPMNIGBM_00913 | #N/A | X:OPMNIGBM_00913:p.His44Asn | #N/A | #N/A | #N/A | #N/A | #N/A |
| Arylsulfatase | CDS: OPMNIGBM_00351, Gene: OPMNIGBM_00351 | X:OPMNIGBM_00351:c.750T>C | #N/A | X:OPMNIGBM_00351:c.750T>C | #N/A | #N/A | #N/A | #N/A |
| Heat-inducible transcription repressor | Gene: hrcA, CDS: hrcA | X:OPMNIGBM_00988:c.954C>A | #N/A | X:OPMNIGBM_00988:c.954C>A | #N/A | #N/A | #N/A | #N/A |
| 50S ribosomal protein L1 | Gene: rplA, CDS: rplA | X:OPMNIGBM_01084:p.Asn34Lys | #N/A | X:OPMNIGBM_01084:p.Asn34Lys | #N/A | #N/A | #N/A | #N/A |
| Hypothetical Protein | Gene: OPMNIGBM_02076, CDS: OPMNIGBM_02076 | X:OPMNIGBM_02076:p.Arg6Lys | X:OPMNIGBM_02076:p.Arg6Lys | X:OPMNIGBM_02076:p.Arg6Lys | X:OPMNIGBM_02076:p.Arg6Lys | X:OPMNIGBM_02076:p.Arg6Lys | X:OPMNIGBM_02076:p.Arg6Lys | X:OPMNIGBM_02076:p.Arg6Lys |
